# Next-Generation Sequencing in Clinical Oncology: Next Steps Towards Clinical Validation

**DOI:** 10.3390/cancers6042296

**Published:** 2014-11-18

**Authors:** Nigel C. Bennett, Camile S. Farah

**Affiliations:** 1 The University of Queensland, UQ Centre for Clinical Research, Herston Qld 4029, Australia; E-Mail: n.bennett@uq.edu.au; 2 The Australian Centre for Oral Oncology Research & Education, Brisbane Qld 4000, Australia

**Keywords:** massively parallel sequencing, next generation sequencing, clinical oncology, validation

## Abstract

Compelling evidence supports the transition of next generation sequencing (NGS) technology from a research environment into clinical practice. Before NGS technologies are fully adopted in the clinic, they should be thoroughly scrutinised for their potential as powerful diagnostic and prognostic tools. The importance placed on generating accurate NGS data, and consequently appropriate clinical interpretation, has stimulated much international discussion regarding the creation and implementation of strict guidelines and regulations for NGS clinical use. In the context of clinical oncology, NGS technologies are currently transitioning from a clinical research background into a setting where they will contribute significantly to individual patient cancer management. This paper explores the steps that have been taken, and those still required, for the transition of NGS into the clinical area, with particular emphasis placed on validation in the setting of clinical oncology.

## 1. Introduction

On 19 November 2013, the U.S. Food and Drug Administration (FDA) approved the use of four diagnostic devices comprising two cystic fibrosis assays, kit reagents and the Illumina MiSeqDx platform for high throughput gene sequencing, commonly known as next-generation sequencing (NGS) [1]. This decision by the FDA has paved the way for future clinical diagnostic and prognostic use of NGS in a multitude of disease settings, including cancer.

Compelling evidence supports the transition of NGS technology from a research environment into clinical practice. While our knowledge of the relationship between disease pathogenesis and genetic variations continues to expand, the cost to NGS equipment and operation continues to fall. Ultimately this will enable relatively cheap exploration of specific nucleic acid regions, large gene panels, whole exomes, genomes, transcriptomes and the methylome. The expansion and acceptance of NGS technologies in many specialty fields of biological research is providing the evidence and promoting its incorporation into the clinical setting. But before NGS technologies are fully adopted in the clinic, they must be thoroughly scrutinised for their potential as powerful diagnostic and prognostic tools. Due to the massive amounts of data generated from patient genetic material, the potential for detailed and extensive analyses is vast. The importance placed on generating accurate NGS data, and consequently appropriate clinical interpretation, has stimulated much international discussion regarding the creation and implementation of strict guidelines and regulations for NGS clinical use.

Establishment of a validation process of current systems for any NGS facility must be undertaken before sample collection and nucleic acid extractions are initiated. This paper is aimed primarily at clinical diagnostic and/or prognostic use of NGS technologies and not the use of NGS equipment for research purposes. In the context of clinical oncology, NGS technologies are currently transitioning from a clinical research background into a setting where they will contribute significantly to individual patient cancer management.

In this paper, we have chosen to present material that we consider important in the field of oncology generally, and also applicable for head and neck cancer (HNC) in particular as our area of interest and expertise. In considering a venture into clinical NGS diagnostics/prognostics, we strongly advise consideration of all information presented herein, and contact with local authorising and monitoring organisations before establishing such workflows.

## 2. Recommendations and Guidelines for Clinical NGS Applications

Internationally, many guidelines and recommendations for regulating and standardising NGS technologies for clinical use have been produced by government, clinical and research organisations (Table 1). The documents listed in Table 1 discuss NGS issues ranging from ethical considerations and patient education through test development and bioinformatics pipeline validation to clinical reporting and data storage. Some documents discuss the entire process comprehensively (*i.e.*, 1, 3, 10) while others focus more on specific areas or relay legal and/or recommended requirements (*i.e.*, 5, 7, 8, 9 and 3, 10). The New York State Department of Health is one government regulatory organisation that has defined and implemented NGS guidelines for validation, quality control and reporting [2]. In combination with this document, laboratories wishing to undertake diagnostic NGS are required to apply for approval, in relation to each specific NGS assay, for genetic testing and oncology purposes [3]. This legislative approach to clinical NGS diagnostics will most likely be common place in the near future. Other guidelines (Table 1) propose extensive, similar and overlapping approaches to NGS clinical implementation. For readers interested in establishing high quality standardised NGS pipelines, we recommend perusal of the information found in Table 1, with particular attention paid to Groups 1, 3, 8 and 10.

**Table 1:** International guidelines and/or recommendations for NGS use.

Group	NGS Document	References	Current Guideline Status
1. Royal College of Pathologists of Australasia (RCPA)	Standards for Massively Parallel Sequencing in Diagnostic Genetic Testing (in development)	[4]	The recommendations presented in this document do not carry regulatory authority.
2. National Association of Testing Authorities (NATA), Australia	Using recommendations in RCPA document for best laboratory practices	[4]	
3. US Centres for Disease Control and Prevention (CDC). Next-generation Sequencing: Standardization of Clinical Testing (Nex-StoCT) workgroup. (USA)	Next-generation Sequencing: Standardization of Clinical Testing (Nex-StoCT) Workgroup Principles and Guidelines.	[5]	Principles and guidelines designed for NGS incorporation into clinical practice while meeting necessary quality assurance standards [1]
4. U.S. Food and Drug Administration (FDA)	Ultra High Throughput Sequencing for Clinical Diagnostic Applications—Approaches to Assess Analytical Validity, June 23, 2011	[6]	The purpose of the meeting was to discuss challenges in assessing analytical performance for ultra high throughput genomic sequencing-based clinical applications.
5. Dutch Society for Clinical Genetic Laboratory Diagnostics (VKGL)	Best Practice Guidelines for the Use of Next-Generation Sequencing Applications in Genome Diagnostics: A National Collaborative Study of Dutch Genome Diagnostic Laboratories	[7]	
6. Whole Genome Analysis group of the Association for Molecular Pathology	Opportunities and Challenges Associated with Clinical Diagnostic Genome Sequencing: A Report of the Association for Molecular Pathology	[8]	
7. American College of Medical Genetics (ACMG)	ACMG clinical laboratory standards for next-generation sequencing	[8]	
8. Wadsworth Centre; New York State Department of Health	“Next Generation” Sequencing (NGS) guidelines for somatic genetic variant detection	[2]	Governed by the Official Compilation of Codes, Rules and Regulations of the State of New York
9. Association for Clinical Genetic Science (ACGS)	Practice guidelines for targeted Next Generation Sequencing analysis and interpretation.	[9]	
10. Clinical and Laboratory Standards Institute (CLSI)	Nucleic Acid Sequencing Methods in Diagnostic Laboratory Medicine; Approved Guideline—Second Edition	[10]	

## 3. Summary of Information Available within Table 1

*Platform selection*: A selection of the documents listed introduces sequencing technologies, NGS platform differences, characteristics and data generation. These documents also cite more detailed information and together can aid appropriate platform selection for a specific NGS purpose [5,7,8,9,11]. *Terminology*: Organisations such as CLSI (Clinical and Laboratory Standards Institute) and ISO (International Standards Organisation) have aligned the use of terminology and definitions to achieve uniformity for global application of NGS standards and guidelines [10]. *Clinical/ethical considerations*: To accurately determine the clinical utility, the relevant and associated benefits of a specific medical intervention, of NGS in patient care a myriad of issues need careful consideration. These include an individual’s clinical presentation, the rationale for a specific NGS test, validation and standardisation of the pipeline and clinical understanding and accurate interpretation of data [8]. Ethical and legal issues such as informed consent, education regarding possible outcomes, confidentiality, sharing of data and counseling services must be addressed by clinicians and NGS facilities prior to test application. Guidelines and recommendations for the handling of specific ethical and legal issues associated with NGS and/or genetic testing are designed to ensure the quality of services and the safety of patients are upheld [4,8,12]. *Sample handling*: To ensure optimum results and to maximise patient benefit, sample collection must be appropriate for the intended test, be stored/transported appropriately to maintain sample integrity and be well labeled for patient identification [10]. *Test development/quality*: At varying levels of detail, all documents listed discuss development and application of NGS tests. Issues covered include test optimisation, wet lab and *in silico* methods validation, detection limit considerations for a specific test and ensuring a robust process are developed. To maximise data quality metrics such as sensitivity, specificity, accuracy and precision associated with downstream informatics pipelines, standard and validated operating procedures must be implemented within NGS laboratories. To this end, all stages of sample processing (*i.e.*, nucleic acid extraction, library preparation, barcoding, target enrichment) inclusive of reference materials (control samples) must be approached in this manner. Routine quality control and quality assurance processes must be maintained and recorded and accreditation with a regulatory organisation sought to guarantee appropriate NGS application and proficiency testing [2,4,5,7,8,9,10,11]. *Test selection*: In consultation with the patient and genetic counselor, the clinician and pathologist will determine which specific analytical approach is relevant to assess a specific disease phenotype. The scope (*i.e.*, distinctions between targeted and non-targeted pathology testing and research studies) of a specific test and intended target region/s (*i.e.*, single gene, gene panel, whole exome, whole genome) need to be clearly defined and be developed from sound peer-reviewed evidence [4]. *Turn-around time*: Each NGS facility should have policies describing acceptable turn-around times based on the specific requirements of each test and clinical importance [11]. *Bioinformatics*: The bioinformatics pipeline contains many separate processes which are designed to ultimately yield useful clinical information from the raw sequencing data. Validation of data output from software algorithms can be achieved by using reference sequences, control samples containing targeted variants and alternative platform/technology confirmation. For variant calling the bioinformatics pipeline relies upon a predefined set of filtering criteria that includes unique reads, mapping quality, base position in read, base quality and read coverage at variant sites. These criteria are incorporated to ensure variant calling accuracy and precision are maintained across different platforms and facilities [7]. *Reporting and data sharing*: Reports need to include all variants detected irrespective of whether targeted, incidental, of known or unknown clinical significance, somatic or germline and the reference sequence used. Each variant needs to be clearly annotated (e.g., HGVS nomenclature guidelines [13]) and its clinical significance stated where possible. Reports should state the limitations of each specific NGS test and reasoning for this, and indicate any issues with an individual test. Reporting on the read coverage, base quality scores and confirmatory testing for each targeted variant should be included. To rapidly build knowledge and improve patient care associated with clinical NGS, facilities are also encouraged to share findings on public databases such as ClinVar [2,8,9,10]. *Data systems and storage*: NGS facilities should ensure that computing hardware, software and networks are appropriately maintained, updated and monitored by suitably qualified experts [4]. Under U.S. government Clinical Laboratory Improvement Amendments (CLIA) regulations (Section 493.1105), analytic systems records and test reports must be stored for at least 2 years. If not appropriately regulated, NGS facilities should disclose to clients which file types and for what length of time NGS data is stored. [11] As a minimum, storage of the variant calling output files (e.g., VCF) is essential. Depending upon facility policies and local government regulations, other files such as FastQ, SAM and/or BAM files should also be retained as a re-analysis option in the future along with bioinformatics processing logs [9]. *Facility management*: Due to the complexity of NGS techniques and data interpretation, it is recommended that both reporting and facility management be done by individuals with appropriate training and certification. Certification should require extensive experience in NGS technologies, genetic sequence variation and determining disease causality from NGS data interpretation [11].

## 4. NGS Use in Clinical Oncology

Currently, investigating genetic material in oncology patients targets specific and well characterised mutations known to promote cancer progression. This is done primarily to diagnose which cancer sub-class (driver mutations) is present and consequently determine whether a targeted therapeutic exists. For example, Herceptin treatment in breast cancer patients targets HER2/neu protein over-expression caused by amplification of the HER2 gene [14]. Genetic testing methods for cancer such as Sanger sequencing, *in situ* hybridisation and karyotyping, currently have limited scope and their diagnostic data yield is relatively low. NGS technologies exploit these weaknesses with high-throughput capabilities, and with the ability to screen a patient’s entire genome, transcriptome and methylome in search of abnormalities and alterations; potential cancer driving variants (pCDVs). Individual patient NGS data may yield multiple pCDVs not currently known to be associated with a specific tumour type. This scenario may tempt physicians/oncologists to administer cancer or non-cancer therapeutics in an off-label manner to help patients who are unresponsive or who may have regressed following best advisable current treatment regimens. However, utilising a therapeutic compound in an untested clinical setting (*i.e.*, off-label use) introduces issues associated with dosage, toxicity and efficacy, other than ethical.

Whole genome data is considered the unbiased gold standard for obtaining sequencing data because intra- and inter-genic regions are revealed entirely [15]. Due to the size of targets, depth of coverage is generally less for whole exomes and genomes than for targeted gene or region sequencing. At present, financial constraints generally limit diagnostics, prognostics and theranostics analyses to targeted gene or region sequencing. But whole exome and eventually whole genome sequencing should be considered for analyses of patients with recurrent disease whereby driver variants are not located on targeted panels. Ideally when cost allows, the whole genome, transcriptome and methylome of every patient should be sequenced consensually. Bioinformatic pipelines can then be designed to focus on targeted or prospective analyses.

To achieve an appropriate quality of care, international oncology organisations have published guidelines and recommendations (Table 2) for clinical diagnosis, treatment and management of cancer patients [16,17,18,19]. These recommendations are designed to assist practitioners during the clinical decision making process by presenting the best available scientific evidence for disease management. In time, we foresee NGS technologies and guidelines (Table 1) being incorporated into these oncology practice guidelines (Table 2) to provide more comprehensive and advanced oncology management practices.

**Table 2:** International oncology practice guidelines.

Oncology Organisation	Guideline Statement	References
The European Society for Medical Oncology (ESMO)	Clinical Practice Guidelines relating to many cancers. Each guideline includes information related to incidence, diagnostic criteria, staging, risk and treatment plans designed to help oncologists deliver an appropriate quality of patient care.	[16]
The National Comprehensive Cancer Network (NCCN)	Treatment guidelines, for most cancers, to improve the quality, effectiveness, and efficiency of cancer care. NCCN offers a number of programs to help guide clinicians through the decision-making process of cancer management.	[17]
The Cancer Council Australia (CCA)	Clinical practice guidelines that bring together the best available evidence to underpin scientifically-valid recommendations for the prevention and diagnosis of cancer and treatment of cancer patients	[18]
American Society of Clinical Oncology (ASCO)	Guidelines can address specific clinical situations (disease-oriented) or use of approved medical products, procedures, or tests (modality-oriented). Using the best available evidence, ASCO expert panels identify and develop practice recommendations for specific areas of cancer care that would benefit from using practice guidelines.	[19]

### 4.1. Clinical Utility

**Figure 1:**
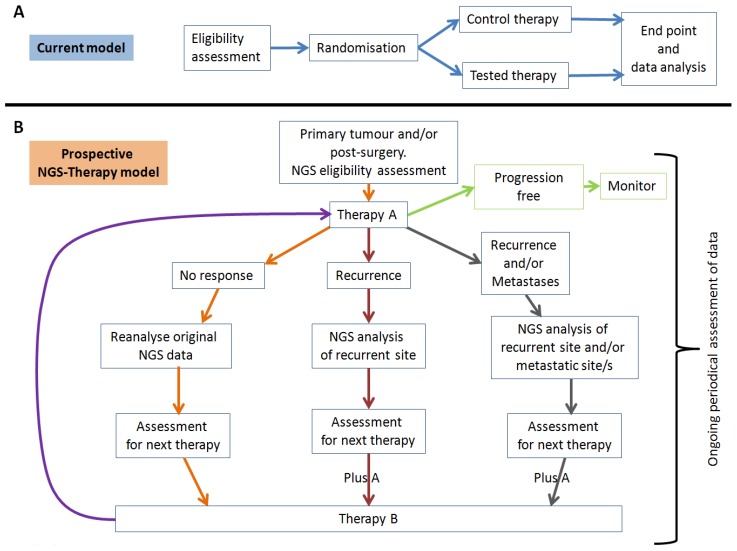
Clinical trials model. *Current model*: standardised protocol, patients are assessed on specific criteria to determine suitability for inclusion; randomised into different therapy groups; trial period is identical for all patients; data analysed retrospectively against control group. *Prospective NGS-Therapy model*: patients, with primary tumour and/or post-surgery, assessed by NGS analyses to determine suitability for inclusion; initially all patients receive the same best-applicable therapy; “no response”, “recurrence” and “recurrence and/or metastases” groups are re-analysed to determine next best-applicable therapy; next therapy for “recurrence” and “recurrence and/or metastases” group may be combined with original “therapy **A**”; “therapy **B**” may or may not be identical for each arm; “therapy B” is recycled into “therapy **A**” position at the top of the flow diagram. Periodical assessment of all trial data is ongoing and updated at each assessment stage.

Suggestive relationships between genotype, phenotype and potential therapies, mainly reported by academic biomedical researchers are generally insufficient for broad clinical application. Integration of NGS technologies into the clinical realm has the capacity to quickly verify suggestive relationships and help remove ambiguity. Reduced turnaround times, decreased sequencing costs and improvements to analysis algorithms are collectively driving NGS technologies in this direction. Initially, to transition NGS technologies into the clinical setting, current accepted genetic tests used for disease diagnoses must be undertaken routinely and successfully with NGS. Subsequently, NGS should be implemented into the current clinical trials model (Figure 1A) to assist retrospective studies in understanding why some patients respond to specific therapies while others do not. Finally, to unlock the full potential and clinical utility of NGS technologies, “suggestive relationships” can be interrogated in prospective NGS-therapy clinical trials (Figure 1B). For NGS technologies to become embedded in clinical practice, they must be rigorously tested for regulatory approval, accepted as routine diagnostic tools, and implemented into clinical trials to ultimately improve patient outcomes [20].

Appropriate implementation of NGS as a clinical diagnostics, prognostics and theranostics oncology tool requires application in clinical trials to expose strengths and weaknesses of current NGS and clinical approaches. We believe one major difference should exist between current clinical trials models and future NGS oncology trials. Once established, these trials should be maintained indefinitely (Figure 1B), with periodic assessment of data for updating the relationship between tissue of origin, cancer sub-type, cancer heterogeneity, pCDVs, therapeutics and patient outcomes. Ideally, what may begin as multiple individual oncology NGS trials may evolve into a collaborative monitoring service for all cancers. Additionally, consensual sampling of metastatic and progressive sites at autopsy may be included for retrospective analyses. Of considerable importance, for use as reference data sets, are the intended ~25,000 cancer genomes, transcriptomes and methylomes readily available from the International Cancer Genome Consortium [21]. This consortium is paving the way for future clinical-NGS applications focused on understanding and treating individual cancer patients.

### 4.2. Tumour Heterogeneity

Initially, neoplastic mutations may be clonal, but divergent sub-clonal mutations accumulate over time and contribute to heterogeneity in all tumours, both primary and metastatic [22,23,24]. Four classes of tumour heterogeneity have been proposed considering spatial, temporal and patient origins; intratumoral, intermetastatic, intrametastatic and interpatient [25]. Heterogeneity analyses of surgically removed primary tumours may be required when either local recurrence occurs due to undetected tumour cells in surgical margins or neighbouring dysplastic tissues progress to primary carcinoma, or when an unrelated primary carcinoma develops in the same tissue due to field cancerization [26]. If metastases are present, the heterogeneic extent of divergence is of great clinical importance. NGS investigation from multiple sites within the excised primary tissue may help generate a tumour specific phylogenetic tree. Mutations common to all tumour sub-clones in the primary site and distant metastases will be located on the trunk of the tree. Initial therapeutic targeting of these combined mutations may provide the basis of effective clinical outcomes [25,27].

### 4.3. Variant Confirmation

Variations (*i.e.*, single nucleotide polymorphisms, insertions, deletions, copy number variations, gene fusions, chromosomal rearrangements), whether germline or somatic found by NGS technologies in cancer samples should be compared to a clinical control sample originating from the same patient where possible. In clinical cancer pharmacogenetics trials, germline DNA samples are typically collected from blood or buccal mucosa [28]. For HNC, patient control samples (reference DNA) must be collected via blood as buccal mucosa samples are unsuitable due to field cancerization [29]. If patient control samples are not available, a reference sequence such as 1000 Genomes Project, dbSNP, HapMap Project, NHLBI Grand Opportunity Exome Sequencing Project, GATK Resource Bundle, NCBI RefSeq, NCBI ClinVar is needed [30].

Considering that thousands of variants can be discovered from a relatively small panel of targeted genes [31], confirmation of all is not clinically plausible nor financially realistic. Confirmation also depends on the specific NGS assay (single *vs.* multiple gene panels) and perceived clinical relevance of specific variations highlighted in NGS data. Regardless of whether the genetic foundation of disease is monogenic (e.g., cystic fibrosis) or polygenic (e.g., asthma or cancer), at present the gold standard for variant confirmation is done using Sanger sequencing. This approach negates the significant number of false-positives currently generated by NGS [32]. An alternative NGS platform can be utilised for confirmation but financial constraints will most-likely shape this decision. In future, Sanger sequencing or alternative platform confirmation processes may be eliminated as NGS technologies improve, error rates decrease and as quality control and proficiency testing systems are implemented and made mandatory. Improvement of current variant-calling algorithms and pipelines are also critical in this arena. A variant, highlighted as being a pCDV, is more likely to be correctly identified if called by multiple algorithms than any one caller [15]. Using multiple variant caller algorithms is a broadly used approach which attempts to mitigate false negative and/or false positive errors [33,34]. Although optimal combinations of variant calling algorithms with respect to specific variant types are not currently known, they would make a welcomed addition to the field [15].

Nucleic acid sequence variation can potentially be targeted or labeled as a disease biomarker. Variation exists in the genome (sequence variant, structural alteration), the transcriptome (splice variant, relative expression) and the methylome (variable methylation signature). Irrespective of their nature, biomarkers need to be readily detectable (with robust technology and validated methods), correlate with a specific tumour or clinical response (have clinical validity and functional relevance), and be exploitable for improved patient survival (have clinical utility via therapeutics) [35]. It has been surmised that sequence variations in cancer can be loosely categorised as either “drivers” (*i.e.*, cancer causing) or “passengers” (*i.e.*, secondary mutations caused by genome instability) [36]. Nonetheless, disease progression and treatment regimens may alter the roles of individual variants [37]. Further sub-classification of “drivers” into a deleterious impact scale (*i.e.*, most important target/s), combined with confidence of clinically relevant interpretation, and application of known drug targets confound the true value of NGS data. Variant confirmation is important and complex but it does not exist in isolation. For reporting purposes all observed pCDVs in processed data should be declared, but only some observed pCDVs will progress to the confirmation phase while others will be eliminated. Justifying the selection process will be difficult when considering the potential clinical impact on the cancer patient. Nonetheless, as confidence within variant-calling algorithms improves interpretation, and increased knowledge of variant impact improves target allocation, coupled with expansion of the pool of therapeutic utilities, difficulties associated with this process will most likely subside.

### 4.4. Reporting and Structure

NGS report structure and item inclusions should follow the reporting requirements of local accreditation and/or regulatory bodies within each state/country and be constructed in consultation with NGS service providers, genetics specialists/counsellors, pathologists, bioinformaticians, pathology service management and clinicians. However it is important that specific details are included in NGS reports such as, but not limited to; gene/chromosomal region interrogated (identified by HUGO Gene Nomenclature Committee guidelines [38]), reference sequence and version number, positions of variations (DNA and amino acid) and predicted effect described as per HGVS guidelines [13], clinical relevance if available, limitations of assay and interpretations, recommendations of additional testing on patient samples or other family members and relationships between variant/s and therapies or prognoses. We suggest a one to two page primary clinical NGS report be produced containing the most pertinent clinical information for immediate review by the requesting medical professional. It is imperative that an extensive and detailed report accompany the primary report which discusses other variants (*i.e.*, incidental findings, variants of unknown significance) and the sequencing, bioinformatics and interpretation workflows and their limitations. Three excellent examples of clinical NGS reporting are presented as additional files by Brownstein *et al.* 2014 [30]. All rare variants should be annotated [36], and detailed descriptions of each reported variant should follow broadly accepted nomenclature guidelines such as those developed by HGVS [9,10,37]. A three tier genetic variant classification system has been proposed for clinical interpretation of NGS data; pathogenic (positive), benign (negative) and VUS (variant of unknown clinical significance) [9,39]. The American College of Medical Genetics (ACMG) and CLSI support a more detailed clinical reporting system that comprises six categories [10,40]. Variant classification however should not be carried out in isolation. NGS data interpretation should be combined with, and considered in light of, other diagnostic tests, physical presentation, clinical history and familial disorders [10]. To this end, it is imperative that bioinformaticians and physicians work together closely.

A recent report by a National Institutes of Health initiative, describes clinical reporting approaches to whole exomes and genomes by six highly regarded NGS specialist consortium members awarded funding to complete a clinical genomic trial with a unique patient cohort. Although this investigation found commonality in sequencing approaches, significant diversity within bioinformatics tools, variant annotation, variant categorisation and structure of final reports was observed. At present there is no user-friendly or standardised course of action for processing sequencing data into a clinical report containing relevant but also correctly identified mutations/variants. Valuable information focussed on this issue can be found in multiple reviews [41,42,43].

### 4.5. Reporting: “Incidental Findings”

As of January 2014, the New York State Department of Health guidelines (Table 1) for somatic genetic variant detection using NGS, state “we suggest you include these (*i.e.*, incidental findings) on your report separately and alert the treating physician to their potential clinical relevance” [2]. The guidelines created by the Royal College of Pathologists of Australasia (RCPA) recommend NGS service providers should develop disclosure policies for reporting of incidental findings. The RCPA advises that NGS service providers inform clinicians and patients about the details of their incidental finding policies and ensure clients understand the nature of the incidental findings reported. Additionally, patients should have the freedom to choose which results they receive. However, exceptional circumstances may require extra management and consultation between clinicians and NGS service providers prior to issuing patient reports. The RCPA advises that where uncertainty arises from reporting of incidental findings, guidance from a medical genetics specialist should be obtained. To minimise unnecessary stress and confusion to patients and their families, disclosure of incidental findings is best limited to variants confirmed as pathogenic [4].

The American College of Medical Genetics and Genomics (ACMG) state that incidental findings are “results that are not related to the indication for ordering the sequencing but may nonetheless be of medical value or utility to the ordering physician and the patient”. The AMCG acknowledge that there is insufficient evidence relating to the benefits, risks and costs of reporting incidental findings to make “evidence-based recommendations”. Keeping this in mind, the ACMG agree that “reporting some incidental findings would likely have medical benefit for the patients and families of patients undergoing clinical sequencing”. Based on these statements, the ACMG has presented a “minimum list” of incidental findings, representing 56 different genes and 24 different groups of disease phenotypes. Excluded from the “minimum list” are diseases primarily caused by structural rearrangements, intra-gene repeat expansions and copy number variations because exome and targeted sequencing cannot reliably produce the data required to accurately confirm such variants [44]. A recent report, following the ACMG guidelines, examined exomes within genomic DNA extracted from peripheral whole blood from 159 families consisting of 543 individuals (188 affected, 137 unaffected, 218 parents). Within the “minimum list” of 56 ACMG recommended genes, 5,948 variants of sufficient quality were confirmed. After annotating and filtering, 250 of these variants were listed as “known pathogenic or probable pathogenic”. Further literature searches to find supporting evidence for classifying these 250 variants as pathogenic resulted in three variants confirmed as “expected pathogenic” and 11 as “known pathogenic”. These 14 variants were present in 5.0% (27/543) of individuals studied (8.8% of families) and no more than one variant was present in an individual or family [45]. The reportable rate for incidental findings in this study was higher than other similar studies (1%–3%) and the ACMG’s estimated rate (1%) [44,46,47]. However, rate discrepancies may be accounted by project differences such as quality and coverage of exomes and differences in variant annotation processes. Adding to these issues, the original authors found compiling evidence for “pathogenicity or probable pathogenicity” was the most time consuming and subjective aspect in the reporting process because “no well-curated comprehensive public database is currently available” [45].

### 4.6. Reporting: Pertinent Negatives

The accurate reporting of a true negative result is equally important as reporting a clinically relevant true positive finding. Verifying that a somatic variant does not exist in a heterogeneic tissue with low tumour cellularity poses the problem; what depth of coverage is needed to confirm a true negative result? The potential negative impact to patient and family members if they receive a false negative result for targeted NGS may be significant. Confidence in reporting a true negative result depends strongly on the sample type being examined. For example, confidence associated with confirming that a mutation does not exist in a pseudo-germline sample (*i.e.*, blood) is significantly greater than confirming the same somatic variant within a tumour of <10% cellularity. To overcome the latter, variant confirmation using defined assay parameters can be achieved. These parameters include, but are not limited to, specifically targeting phenotype-associated genes to achieve a high overall depth of coverage (e.g., >500×), increasing tumour cellularity if possible, ensuring quality control standards are met during all NGS processes and including both positive and negative control samples for the targeted variant. If any one of these pre-determined parameters is considered inferior during bioinformatics analyses, the NGS assay must be repeated to obtain confidence for reporting and ensures the NGS service provider is adhering to the principles of good laboratory practice.

### 4.7. Clinical Bioinformatics

The purpose of bioinformatics, variant interpretation and reporting of NGS data is to provide meaningful and explicit information to clinicians, patients and their families. Created by the Boston Children’s Hospital, The Children’s Leadership Award for the Reliable Interpretation and appropriate Transmission of Your genomic information (CLARITY) Challenge was designed to identify best methods and practices to this end. Thirty international bioinformatics groups were solicited to interpret and report on the same NGS data from 12 participants in three different families with heritable genetic disorders. The sequencing data consisted of whole exomes (SOLiD 5500xl) from all 12 participants and whole genomes (Complete Genomics Incorporated) from 10 of the participants. Families 1 and 3 consisted of a child (proband) and both parents while family 2 consisted of two first cousins (probands) and both sets of parents. All 30 bioinformatics groups were given the same clinical diagnoses and family histories for all three families. The groups were asked to analyse and interpret the NGS data then report the disease causing variants identified. Twenty-three groups submitted reports. By consensus, five specific genes contained clinically relevant or plausible disease causing variations in these three families. On average, approximately one third of groups reported any one of these mutations. Although the challenge was completed admirably by some groups, the amount of disparity in the cumulative results is concerning. The 2014 report highlights similarities in bioinformatics techniques, but interpretation and reporting revealed low concordance [30]. Possible causes for divergence amongst the groups included limited quality control measures undertaken prior to variant calling, different read aligners used by different groups, different variant calling pipelines as a source of significant variability in analyses outcomes, variations in performance of GATK and SAMtools associated with sequencing depth, clinically relevant variants being missed by calling software, variants eliminated by downstream filters, variants manually eliminated (due to lack of clinical knowledge, doubted published literature, considered irrelevant, VUS, identified but ignored in favour of another), programming errors, and poor quality data (low coverage and noisy location) [30,48,49]. Successful identification of the small number of causative genetic variants for each phenotype was not achieved by the majority of participating bioinformatics groups. Although the diseases analysed in this challenge were not cancers, the bioinformatics and interpretation discrepancies highlighted are very likely to be further compounded with analyses of polygenic, heterogeneic diseases such as cancer.

## 5. Conclusions

Analysing clinical cancer samples with NGS is proceeding along two discrete paths; multiple sample exploration (research based) and single sample diagnostics for prognostic purposes (specific clinical purpose). Over time, both paths have the capacity to influence and benefit one another, but validation (platform, targets and bioinformatics pipeline), quality control (monitoring of accuracy, precision, operator, platform, assay targets and bioinformatics pipeline over time), result reporting and proficiency testing (periodic testing of facilities by third parties) may not be equally relevant or similarly implemented in both categories. This is primarily due to differences in expected levels of quality control (e.g., minimum coverage, base quality scores) and accreditation differences between research and diagnostics facilities [50].

A recent report from the American Society of Clinical Oncology (ASCO) emphasizes the increasing number of cancer patients, increasing cancer care costs, increasing disparity of patients to cancer care workers (e.g., oncologists, nurses) and increasing therapeutic co-morbidities [51]. Introducing NGS technologies into the current oncology setting has great potential in alleviating and reducing these issues in the future. Although still relatively expensive for individual patient use, especially if whole exomes or genomes are investigated, continued development in this field is driving costs down. This, combined with greater potential for multiplexed patient samples per run, will lead to wider spread application in growing numbers of patients in the near future. Widespread application of NGS technologies in the clinical setting will generate enormous amounts of valuable data specific to each cancer type and causal variants. In turn, this resource will help to elucidate driver and passenger mutations, better monitor the efficacy of therapeutics, increase survival rates and reduce induced co-morbidities. As this wealth of NGS-cancer specific information grows, valuable clinical-research information will flow back to the oncologist and ultimately to the beneficiary, the cancer patient, in the form of contemporary, personalised, therapeutic strategies.
